# Correlation between white matter hyperintensity and delusional symptoms in Alzheimer’s disease

**DOI:** 10.1186/s12888-023-05420-5

**Published:** 2023-12-06

**Authors:** Wei Fan, Shaolun Ma, Ziqi Wang, Yuanyuan Han, Xiaowei Liu, Rui Gu, Qingyan Cai

**Affiliations:** 1 The Fourth People’s Hospital of Chengdu, Chengdu, China; 2 University of Electronic Science and Technology of China, Chengdu, China

**Keywords:** Alzheimer’s Disease, Delusional symptoms, White matter hyperintensity

## Abstract

**Background:**

Patients with Alzheimer’s disease (AD) often exhibit neuropsychiatric symptoms (NPS), particularly delusions. Previous studies have shown an association between white matter hyperintensities (WMH) and specific NPS. This study aims to explore the relationship between WMH volume and delusions in AD patients by comparing the WMH volumes of delusional and non-delusional subgroups.

**Methods:**

80 AD patients were divided into a delusion group (n = 36) and a non-delusion group (n = 44) based on the Neuropsychiatric Inventory (NPI). The brain cortical volume and WMH volume were quantitatively calculated for all 80 patients, including total WMH volume, periventricular WMH (PVWMH) volume, deep WMH volume, as well as bilateral frontal lobe, temporal lobe, parietal lobe, and occipital lobe WMH volumes. Firstly, we compared the differences in WMH volumes between the delusion group and non-delusion group. Then, within the delusion group, we further categorized patients based on severity scores of their delusional symptoms into mild (1 point), moderate (2 points), or severe groups (3 points). We compared the WMH volumes among these three groups to investigate the role of WMH volume in delusional symptoms.

**Results:**

There was a significant difference in left occipital lobe WMH volume between the delusion group and non-delusion group(*P* < 0.05). Within the delusion group itself, there were significant differences in overall WMH volume as well as PVWMH volume among patients with mild or severe levels of delusions(*P* < 0.05).

**Conclusion:**

Left occipital lobe WMH volume may be associated with the occurrence of delusional AD patients, and the total volume of whole-brain WMH and PVWMH volume may affect the degree of severity of delusional symptoms.

**Supplementary Information:**

The online version contains supplementary material available at 10.1186/s12888-023-05420-5.

## Introduction

Alzheimer’s disease (AD) is a neurodegenerative disorder characterized by progressive memory decline, accompanied by other cognitive impairments, neuropsychiatric symptoms, and personality changes. It is the leading cause of dementia and rapidly becoming one of the most expensive, deadly, and burdensome diseases of this century [1]. Nearly all AD patients experience neuropsychiatric symptoms (NPS) [2]. NPS refers to non-cognitive mental and behavioral symptoms observed in dementia patients, involving perception, emotion, behavior, etc., mainly including hallucinations, delusions, depression, apathy [6]. Studies have found that the prevalence of NPS in dementia ranges from 50 to 80% [3], and NPS may occur before cognitive decline [4]. Some studies suggest that specific NPS may contribute to early prediction or onset of dementia [5]. NPS can exacerbate cognitive symptoms and functional decline while increasing the risk of mortality for AD patients [7], with limited treatment options available [8], although psychotropic drugs can temporarily alleviate certain NPS symptoms [9, 10], however some medications have significant adverse effects.

The etiology of NPS in AD remains unclear but there is evidence suggesting an association between focal white matter hyperintensities (WMHs) and NPS [11–13]. WMHs are a type of cerebral small vessel disease characterized by damage to the brain’s white matter. They were first proposed by Hachinski et al. as common radiological findings on neuroimaging examinations. On T1-weighted imaging in magnetic resonance imaging (MRI) [14], WMHs appear as iso- or hypointense signals; on T2 Flair-weighted imaging they appear as punctate or patchy lesions. WMH burden has been associated with AD pathology and it is speculated that vascular-originated WMHs are also related to NP Ssymptoms including AD [15, 16]. The significance of white matter hyperintensities (WMH) in patients with mild cognitive impairment (MCI) and early Alzheimer’s disease (AD) suggests that WMH may not be a secondary pathology of AD, but rather an important factor in the occurrence of neuropsychiatric symptoms (NPS). NPS generation may be based on changes in connectivity between brain regions. In contrast, ischemic lesions may disrupt connectivity between brain regions more significantly than cortical atrophy [9]. Additionally, WMH undergoes changes in the pathological process of AD [17]. In AD patients, WMH can be identified on MRI long before the onset of cognitive impairment or NPS symptoms and may have unique contributions to the clinical manifestations of this disease [18]. Lee et al. found that autosomal dominant AD patients had increased WMH six years before clinical symptoms appeared and discovered a certain correlation between WMH and Aβ levels in cerebrospinal fluid carriers. Previous studies reported a relationship between delusions and severity of white matter changes observed on MRI (Lee et al., 2006) [19].

The pathogenesis of cerebral white matter lesions is still unclear, but most studies believe that deep white matter lesions are mainly caused by chronic cerebral ischemia resulting from small vessel chronic diseases as well as neurofiber demyelination, axonal loss, glial cell proliferation, and parenchymal damage caused by blood-brain barrier injury [20]. Patients with dementia often report multiple NPS, and apathy, depression and anxiety are frequently found to be the most commonly reported symptoms in these patients [21, 22]. Delusional symptoms are one of the highest burdens that cause pain to caregivers [23]. Research has shown that delusional symptoms in AD patients are associated with more severe functional impairments, malnutrition risk increase, pressure ulcer risk increase, and increased mortality risk. Revised sentence: “Delusions in patients with Alzheimer’s disease (AD) are associated with structural and functional changes in the brain, including alterations in both gray matter and white matter. Previous studies have indicated that WMH observed on MRI of patients with AD is associated with delusions and depression [24]. WMH of the parietal and occipital lobes may impact delusional symptoms in AD patients [25, 26].

However, there is limited research on the role of white matter hyperintensities (WMH) in delusions among AD patients, and existing findings are inconsistent. Therefore, further comprehensive investigations into delusional symptoms among AD patients are necessary. Our study hypothesizes that differences may exist in WMH volume or specific brain regions between AD patients with and without delusional symptoms, aiming to explore potential mechanisms underlying the relationship between delusional symptoms and WMH in AD.“

## Methods

General data: A total of 80 cases of Alzheimer’s disease patients who visited the memory clinic from April 2021 to March 2022 were included in this study. Patient basic information was collected by reviewing case data, including age, gender, education level, occupation, handedness, etc. Additionally, past medical history as well as smoking and drinking history were obtained. The inclusion criteria were as follows: (1) AD diagnosised by the National Institute on Aging- Alzheimer’s Association (NIA-AA) in 2011, with an age of ≥ 50 years old. (2) Having a Magnetic Resonance Imaging hippocampal coronal section MTA score2 points. (3) Being diagnosed as probable Alzheimer’s disease by at least two deputy chief-level psychiatrists or neurologists. (4) Undergoing complete evaluations using MMSE, ADL, NPI scales in the memory clinic and Siemens 3TMR imaging within one week. (5) Reliable caregivers being present during assessments. (6) Approval from our hospital’s ethics committee for conducting this study protocol. (7) Ensuring that patients or their legal guardians were informed about this study and signed informed consent forms.

The exclusion criteria were defined as follows:

(1) Presence of other types of dementia such as vascular dementia(VaD: with a clear obvious dementia syndrome occurring within six months after cerebrovascular events), frontotemporal dementia(FTD), Lewy body dementia(DLB), etc., or presence of diseases that may cause other dementias or cognitive impairments such as head trauma, brain tumors, epilepsy, cerebral inflammatory diseases, demyelinating diseases of central nervous system, normo-pressure hydrocephalus, severe liver or kidney dysfunction, severe anemia, hypothyroidism, vitamin B12 deficiency, and so on. (2) Presence of white matter hyperintensities or other types of white matter lesions resulting from radiation injury, carbon monoxide poisoning, multiple sclerosis (MS), vasculitis, or cerebral white matter malnutrition.(3) Clear history of cerebrovascular disease with a Hachinski Ischemic Score (HIS) > 4 and Fazekas score ≥ 2.(4) Clear history of psychiatric disorders such as schizophrenia or severe depression (HAMD scale score < 8), or clear history of substance abuse including drugs and alcohol.(5) Accompanied by severe disorders, severe aphasia, or inability to complete neuropsychological tests.

Neuropsychological testing: Professional evaluators trained in consistency at the Memory Clinic conduct cognitive assessments of patients. The Mini-Mental State Examination (MMSE), with a maximum score of 30, is utilized. The MMSE scale comprises orientation (10 points), memory (3 points), attention and calculation abilities (5 points), recall ability (3 points), language skills (8 points), and visuospatial skills (1 point). Daily living activities assessment: The Activities of Daily Living Scale (ADL) is utilized, with a total score of 80. This includes the Basic Activities of Daily Living Scale (BADL) consisting of 11 items, and the Instrumental Activities of Daily Living Scale (IADL) comprising 9 items. A higher score indicates poorer daily living activity capabilities. Neuropsychiatric behavior evaluation: Reliable caregivers are interviewed using the Neuropsychiatric Inventory questionnaire(NPI), which covers symptoms such as delusions, hallucinations, agitation/aggression, depression/mood disturbances, anxiety, euphoria/elation, apathy/indifference, disinhibition, irritability/emotional lability abnormal motor behavior, sleep/nighttime behavior, and appetite/eating disorders. Scores are assigned based on frequency and severity of episodes as well as distress caused to caregivers. The total score is 144; higher scores indicate more severe neuropsychiatric behaviors.

According to the NPI scale, a total of 80 patients were categorized into two groups: delusional (n = 36) and non-delusional (n = 44). Among the 36 delusional patients, 12 exhibited mild severity scores (score = 1), while 9 had moderate severity scores (score = 2), and the remaining 15 experienced severe symptoms.

### MRI data acquisition

Equipment: 3.0T Magnetom Syngo Skyra, Siemens, Erlangen, Germany, standard 20-channel magnetic head receiving coil for imaging.

### MRI data analysis

Quantitative WHM analysis of white matter: magnetic resonance imaging scan sequence and parameters Structural magnetic resonance imaging: three-dimensional magnetization was used to prepare rapid gradient echo MP-RAGE sequence. Imaging parameters were as follows: The in-plane resolution was 1mmx1mm, the layer thickness was 1 mm, the total number of layers was 180, FA = 8, TR = 7.1ms, TE = 3.0ms, TI = 900ms, TD = 2400ms, FOV = 24cmx18cm, matrix = 256 × 256. Structural magnetic resonance data preprocessing and analysis: SPM software package was used to register the original structural image to the MNI standard template by affine transformation, and then the registered image was segmented into gray matter, white matter, and cerebrospinal fluid. FLAIR sequences were segmented by automatic WMH segmentation. T1, white matter hyperintensity processing: WMH volume (in MM3) was calculated for the whole brain, paraventricular and deep subcortical regions, and each brain region. The head size was normalized and log-transformed to achieve normal distribution.

**Fig. 1:**
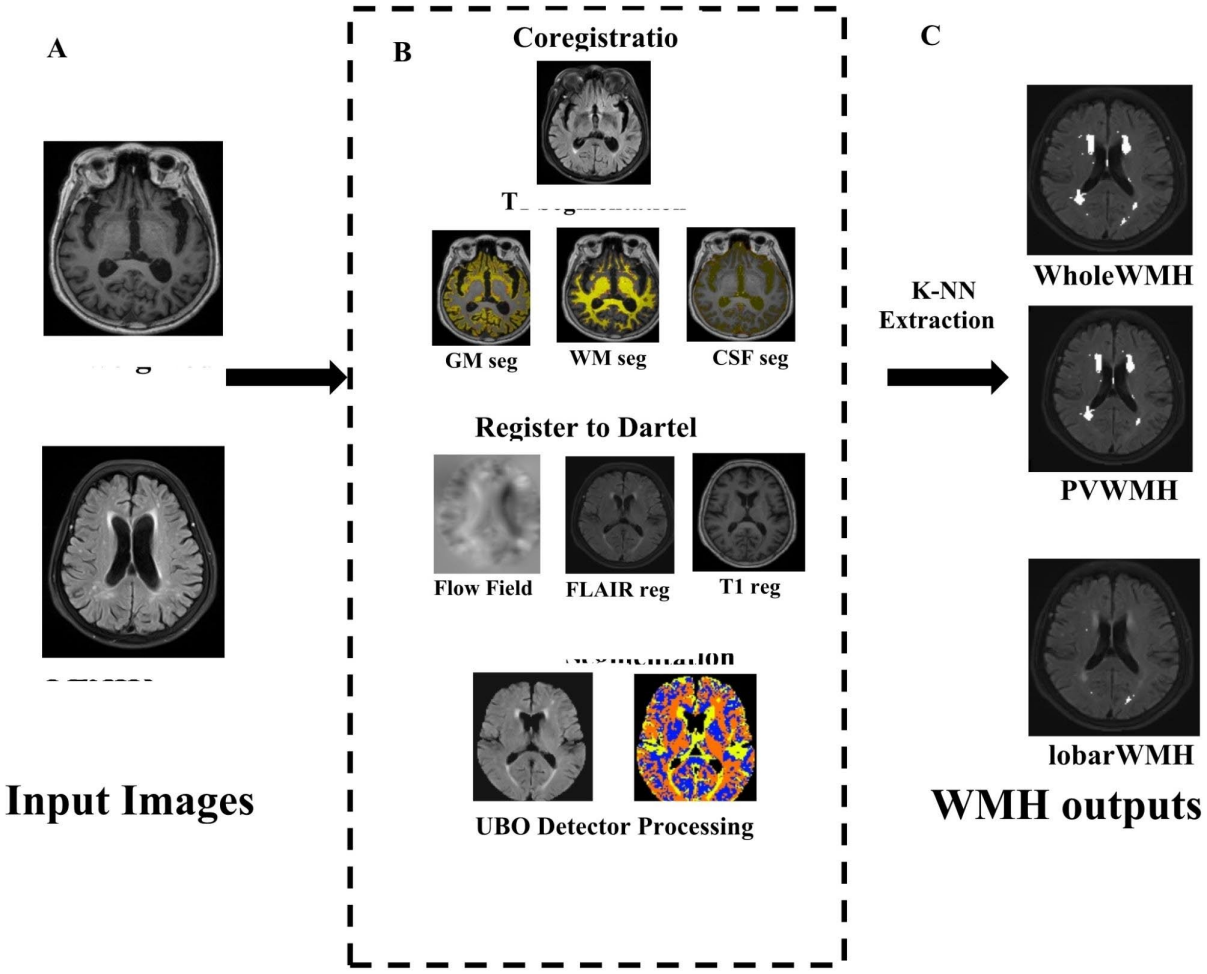
Flowchart of WMH extraction. (**A**) Input the raw T1-weighted and FLAIR images into the UBO detector. (**B**) UBO Detector Processing Pipeline. UBO Detector first performs a rigid-body registration with T1 as the reference and FLAIR as source image and conducts T1 segmentation using SPM12 function. And then the individual T1, coregistered FLAIR, as well as the GM, WM and CSF images were registered to a DARTEL template provided by UBO. Finally, FMRIB’s FAST Segmentation was conducted on skull-stripped and bias field-corrected FLAIR images to generate candidate WMH clusters. (**C**) K-NN algorithm was performed to determine whether the candidate clusters are WMH or non-WMH

### Statistical analysis

①Statistical analyses of all cognitive and questionnaire data were analyzed using SPSS version 21 ②The Shapiro-Wilk test was used to assess if the continuous variables showed signifificant deviations from normality. For variables that did not signifificantly deviate from normality, a two-sample t test was used to evaluate the difference between delusional patients and non-delusional control groups. For signifificantly non-normal data, the Mann-Whitney U test was used. The x2 test of independence was used to statistically assess categorical variables. Signifificance level was set at *p* < 0.05. As demographic and cognitive data were primarily used to assess if the two cohorts were comparable, no corrections were used for multiple comparisons to provide a more conservative test for between-group differences.

## Results

**Fig. 2:**
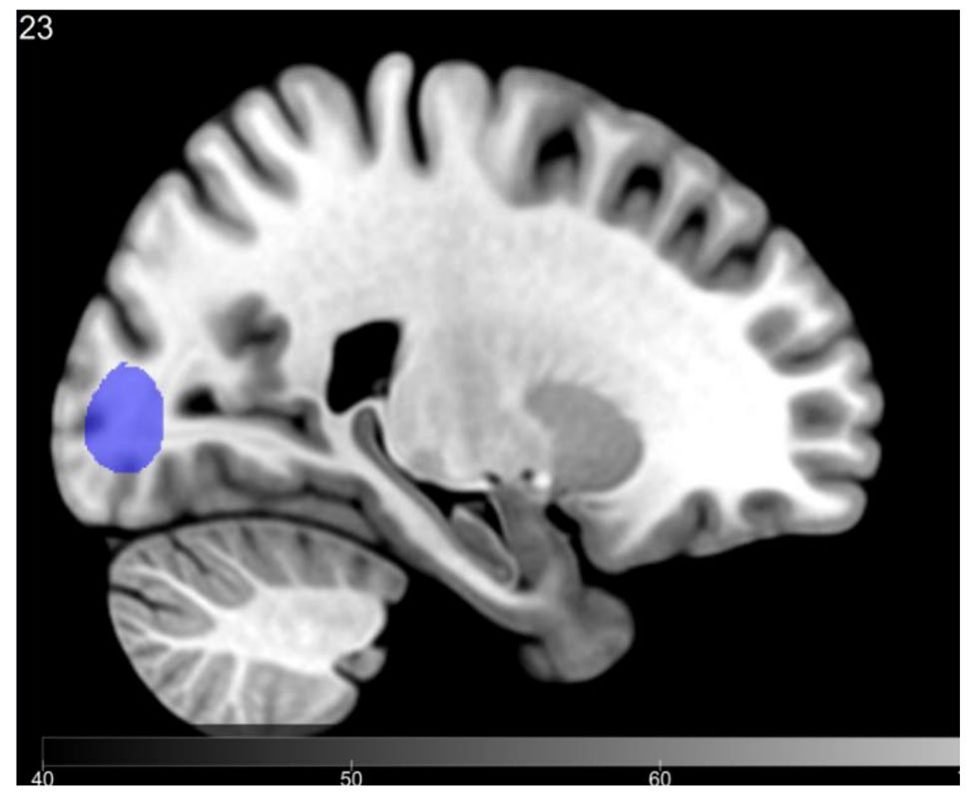
There was a significantly greater WMH volume in the left occipital lobe in those with delusions (p=0.015)

**Fig. 3:**
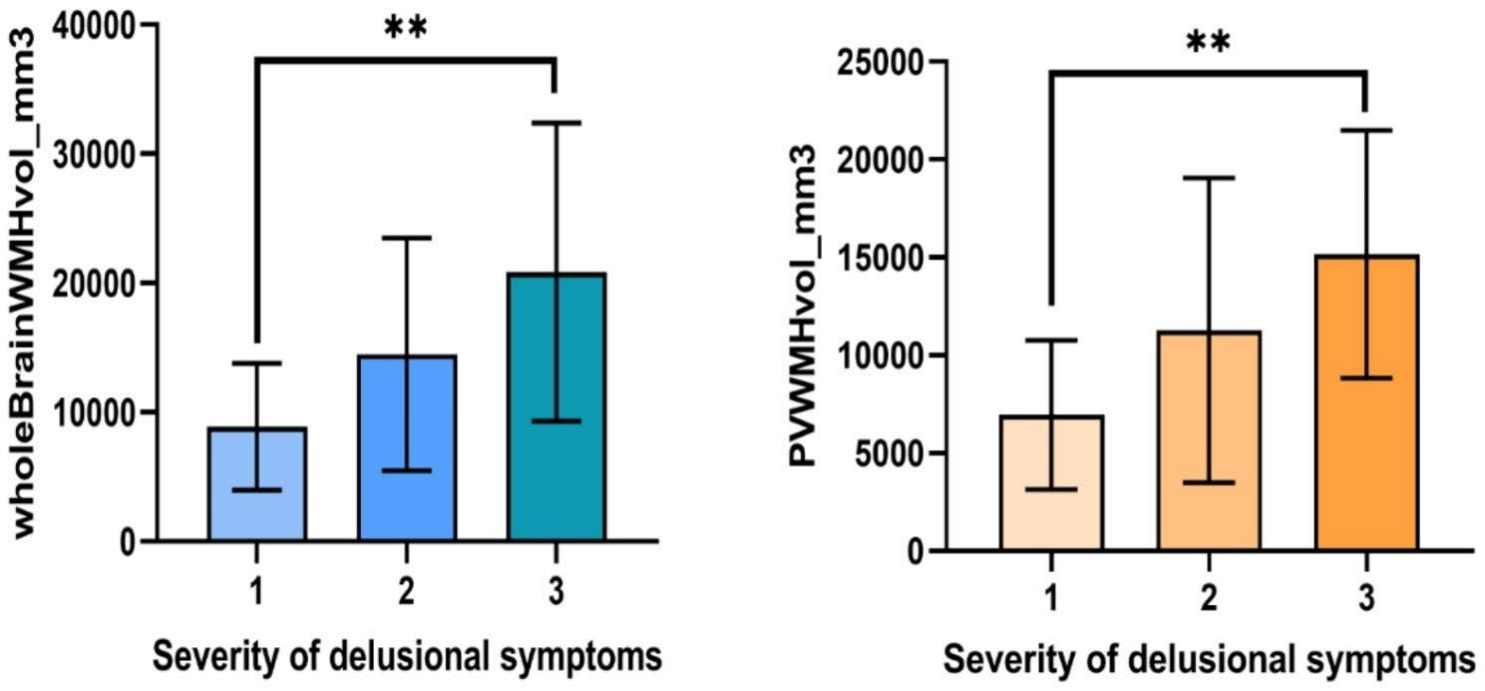
Secondary analysis showing signifificantly serious Whole WMH and PVWMH in delusional subjects with moderate to severe delusions (n = 15) compared with subjects with mild delusions (n = 12). Greater delusional severity was associated with serous White matter damage in the brain(**)

A total of 80 participants were included in this study, and the clinical characteristics of the participants are presented in Table 1. In the AD group, patients with delusions had an average age of 77.19 ± 9.052 years, including 6 males and 30 females. The average duration of education was 7.86 ± 5.405 years. The MMSE total score was 9.33 ± 7.313 points, and the ADL total score was 43.03 ± 15 .609 points. For non-delusional AD patients, the average age was75 .61 ± 10 .292 years, with 11 males and 33 females, and an average education duration of 7 .77 ± 4 .759 years. The MMSE total score was 10 .84 ± 7 .156, and the ADL total score was44 .34 ± 17 .978. There were no significant differences between the delusional and non-delusional groups in terms of gender, age, education duration, MMSE scores or ADL scores. However, the NPI scale total score for the delusional group was33 .14 ± 28 .376, while it was 16 .7 ± 15 .753 for the non-delusional group(*P* = 0 .002), indicating a statistically significant difference. The NPI caregiver distress rating for the delusional group was 15.33 ± 11.089, compared to 7.64 ± 6.65 for the non-delusionaI group(*p* = 0.000), also demonstrating statistical significance. U tests were conducted to compare volumes of high-intensity white matter (WMH) on resonance imaging (MRI) between two groups to examine their relationship with delusion symptoms. In both patient groups, the brain cortical volume, lateral ventricle volume, temporal lobe volume, prefrontal lobe volume, right occipital lobe WMH volume, PVWMH(volume of periventricular white matter hyperintensities), DWMH(volume of deepwhite matter hyperintensities), and total WMH volume did not show any statistically significant differences. However, a statistically significant difference in left occipital lobe WMH volume was observed (*P* < 0.05) (Table 2, Tables 3 and Fig. 2). An analysis of the impact of white matter hyperintensity (WMH) volume on delusion severity in a cohort of delusional patients revealed that Alzheimer’s disease (AD) patients exhibited an exacerbation in delusion severity with increasing WMH burden. To compare NPI delusion severity scores among participants, we conducted a one-way ANOVA, including 12 out of 36 participants with a score of 1, 9 out of 36 participants with a score of 2, and 15 out of 36 participants with a score of 3. The total WMH score for the delusional group showed significant differences (F = 5.716, *P* = 0.007), as did the PVWMH total score (F = 6.14, *P* = 0.005). Post-hoc tests indicated statistically significant differences in both total WMH volume and PVWMH volume mild and severe delusion symptom groups (*P* = 0.004 and *P* = 0.003 respectively) (Table 4 and Fig. 3).

**Table 1:** Clinical and Demographic Data of Delusional and Non-Delusional Participants

	Delusional n = 36	Non-Delusional n = 44		
Variable	mean	mean	Statistical Value	
Sex	6 males	11 males	t = 0.9 *p* = 0.371	
	30 females	33females		
Age	77.19 ± 9.052	75.61 ± 10.292	t = 0.721	*p* = 0.364
Education	7.86 ± 5.405	7.77 ± 4.759	t = 0.078	*p* = 0.938
MMSE	9.33 ± 7.313	10.84 ± 7.156	t=-0.928	*p* = 0.356
ADL	43.03 ± 15.609	44.34 ± 17.978	t=-0.345	*p* = 0.731
NPI total	33.14 ± 28.376	16.7 ± 15.753	t = 3.277	*P = 0.002
NPI caregiver	15.33 ± 11.089	7.64 ± 6.65	t = 3.661	*p = 0.000

Computed using the 2-sample t test

**Table 2:** WMH volume Data of Delusional and Non-Delusional Participants

	whWMH	PVWMH	DWMH	Lfrontal	Rfrontal	Ltempor	Rtempor	Lparieta	Rparieta	Loccipita	Roccipita
Mann-Whitney	668	663	704	736	735.5	702.5	732.5	690	726.5	541.5	692
Wilcoxon	1658	1653	1694	1726	1725.5	1692.5	1722.5	1680	1716.5	1531.5	1682
Z	-1.199	-1.248	-0.851	-0.542	-0.547	-0.866	-0.578	-0.987	-0.633	-2.423	-0.967
sig	0.23	0.212	0.395	0.588	0.585	0.386	0.563	0.324	0.526	0.015**	0.333

**The significance level of the difference between means is <0.05

**Table 3:** GM volume Data of Delusional and Non-Delusional Participants

	GM
Mann-Whitney	719.000
Wilcoxon	1385.000
Z	− 0.706
sig	0.480

**The significance level of the difference between means is <0.05

**Table 4:** Data of different severity within delusional groups

Variable(WMH)				mean(I-J)	SD	sig
whole	Dunnetts t	1	3	-11968.593750**	3553.37756	0.004**
PVWMH	Dunnetts t	1	3	-8204.962500**	2342.430753	0.003**
DWMH	Dunnetts t	1	3	-3751.3125	1886.471757	0.101
Lfrontal	Dunnetts t	1	3	-243	122.777564	0.103
Rfrontal	Dunnetts t	1	3	-169.9875	127.195363	0.326
Ltemporal	Dunnetts t	1	3	-158.45625	123.607895	0.354
Rtemporal	Dunnetts t	1	3	-52.3125	36.887853	0.286
Lparietal	Dunnetts t	1	3	-1275.075	760.764024	0.184
Rparietal	Dunnetts t	1	3	-1246.21875	1041.86245	0.401
Loccipital	Dunnetts t	1	3	-250.03125	149.743302	0.186
Roccipital	Dunnetts t	1	3	-356.23125	244.001226	0.268
Lcerebellum	Dunnetts t	1	3	5.34375	4.139251	0.349
Rcerebellum	Dunnetts t	1	3	-1.8	1.759	0.505

**The significance level of the difference between means is <0.05

## Discussion

Through quantitative analysis of white matter hyperintensities (WMH) volume, we observed that AD patients exhibiting delusional symptoms displayed significantly larger WMH volumes compared to those without delusions. This finding supports our hypothesis suggesting a potential association between the development of delusional symptoms and increased WMH burden. Additionally, we identified a correlation between the occurrence of delusions and WMH volume specifically in the left occipital lobe.

In the realm of psychiatric treatment, it is commonly observed that individuals with AD often present severe delusional symptoms, which significantly impede their daily functioning and impose a substantial burden on caregivers. The findings of this study also demonstrate that AD patients with delusions exhibit notably higher total scores on Neuropsychiatric Inventory (NPI), more pronounced caregiver burden compared to those without delusions. Moreover, there is currently an absence of effective pharmacological interventions for managing delusional symptoms in AD patients, while non-pharmacological approaches frequently prove ineffective. In clinical practice, low-dose antipsychotic medications are frequently employed to ameliorate these symptoms; however, their usage often leads to serious adverse reactions and escalates patient mortality risk. Consequently, it is imperative to promptly identify potential mechanisms underlying delusional symptoms in AD patients and strive towards enhancing their quality of life during advanced stages of the disease from a preventive standpoint while concurrently alleviating patient distress.“

Currently, there is no consensus regarding the underlying mechanism of delusional symptom development. Delusional symptoms can manifest in various diseases such as bipolar disorder, schizophrenia, and Alzheimer’s disease. Studies investigating gray matter (GM) structures have identified relative atrophy in several brain regions among patients with delusional symptoms, including the dorsolateral prefrontal cortex, left putamen, hippocampus, insula, amygdala, thalamus, superior temporal gyrus and middle frontal gyrus. However, the specific affected brain regions vary significantly due to disruption [6, 7] study utilizing data from a large multicenter database initiated by the Alzheimer’s Disease Neuroim Initiative revealed that white matter hyperintensities (WMH) exerted a greater impact on neuropsychiatric symptoms (NPS) compared to focal cortical atrophy. The influence of WMH on NPS may be independent of other factors [10].“

Previous studies have investigated the association between WMH and NPS [23, 27, 28], yet the findings have been inconsistent. In this study focusing on WMH in patients with AD, we compared the differences in WMH volume between patients with delusions and those without delusions. Within the delusional group, we further examined WMH volume based on the severity of delusional symptoms to explore the relationship between WMH and delusions in AD patients. Additionally, we analyzed differences in GM volume between the groups with and without delusions to minimize potential confounding effects of cortical atrophy on our results. Our study revealed a significant difference (*P* < 0.05) in left occipital lobe WMH volume between AD patients with and without delusions. As depicted in Fig. 1, WMH levels were evenly distributed from 0 to 2000 within the delusional group, while they were primarily distributed from 0 to 1000 within the non-delusional group. The average volume of WMH was smaller in the non-delusional group compared to the delusional group. These findings suggest that left occipital lobe WMH may be associated with the occurrence of delusions, partially supporting previous research by Na et al. [26] indicating that occipital white matter changes may contribute to development of delusions in AD patients.1. “In the delusional group, we categorized into mild, moderate, and severe groups based on their severity. The overall volume of white matter hyperintensities (WMH) exhibited a significant difference (F = 5.716, *P* = 0.007), while the total volume of periventricular also showed a statistically significant difference (F = 6.14, *P* = 0.005). Posthoc analysis revealed a significant difference in WMH volume between the mild and severe delusional symptom groups (*P* = 0.004). Similarly, there was a statistically significant difference in PVWMH volume between these two groups (*P* = 0.003), as depicted in Fig. 2 which clearly illustrates an increasing trend of delusional symptom severity with higher WMH volumes. These findings suggest that both total WMH volume contribute to the progression of delusional symptoms.“

## Conclusion

In summary, our findings suggest that the volume of WMH in the left occipital lobe may exert an influence on the development of delusional symptoms. Moreover, in patients with AD, an increase in WMH volume further exacerbates the severity of these symptoms. These results support previous research indicating a potential relationship between WMH and the occurrence and progression of delusions, suggesting that abnormal information pathways resulting from white matter damage contribute to their manifestation. To elucidate the underlying mechanism by which WMH affects clinical manifestations in AD, studies are warranted for future investigation. Currently, there is still insufficient evidence to definitively conclude whether WMH can predict AD conversion; only a few studies have suggested its clinical continuity as a predictor for disease progression [29–31]. It should be mechanisms underlying neuropsychiatric symptoms (NPS) in AD patients are multif, with WMH being just one visible manifestation among them. Future research endeavors should incorporate more reliable biomarkers and multimodal imaging techniques to explore comprehensively not only delusional symptoms but also other psychiatric and behavioral manifestations observed in individuals with AD.“

### Electronic supplementary material

Below is the link to the electronic supplementary material.

**Supplementary Material 1: TABLE S1**. Data of different severity within delusional groups

## Data Availability

The data supporting the conclusions of this article are included within the article.

## Abbreviations

AD: Alzheimer’s disease
DWMH: Deep white matter hyperintensities
MRI: Magnetic Resonance Imaging
NPI: Neuropsychiatric Inventory
NPSs: Neuropsychiatric symptoms
PVWMH: Periventricular white matter hyperintensities
WMH: White matter hyperintensities
