# Supplementary material for: Correlation between white matter hyperintensity and delusional symptoms in Alzheimer’s disease
Source: BMC Psychiatry. 2023 Dec 6;23:914. doi: 10.1186/s12888-023-05420-5 (PMC10698988; doi:10.1186/s12888-023-05420-5)
Supplement: Supplementary file 1 — Supplementary Material 1: TABLE S1. Data of different severity within delusional groups [file 12888_2023_5420_MOESM1_ESM.docx]

| 5.TABLE S1. Data of different severity within delusional groups | | | | | | |
| --- | --- | --- | --- | --- | --- | --- |
| Variable(WMH) |  |  |  | mean(I-J) | SD | sig |
| whole | Dunnetts t | 2 | 3 | -6365.25 | 3868.427505 | 0.195 |
| PVWMH | Dunnetts t | 2 | 3 | -3883.275 | 2550.115601 | 0.241 |
| DWMH | Dunnetts t | 2 | 3 | -2452.125 | 2053.730319 | 0.402 |
| Lfrontal | Dunnetts t | 2 | 3 | -232.125 | 133.663281 | 0.165 |
| Rfrontal | Dunnetts t | 2 | 3 | -79.425 | 138.472772 | 0.799 |
| Ltemporal | Dunnetts t | 2 | 3 | -69.675 | 134.567232 | 0.832 |
| Rtemporal | Dunnetts t | 2 | 3 | 2.25 | 40.158408 | 0.998 |
| Lparietal | Dunnetts t | 2 | 3 | -1101.45 | 828.214966 | 0.329 |
| Rparietal | Dunnetts t | 2 | 3 | -267 | 1134.236171 | 0.962 |
| Loccipital | Dunnetts t | 2 | 3 | -350.625 | 163.019859 | 0.072 |
| Roccipital | Dunnetts t | 2 | 3 | -354.075 | 265.634889 | 0.327 |
| Lcerebellum | Dunnetts t | 2 | 3 | 0 | 4.506246 | 1 |
| Rcerebellum | Dunnetts t | 2 | 3 | -1.8 | 1.915 | 0.559 |
| **The significance level of the difference between means is ＜0.05. | | | | | | |
